# A Narrative and Case-Illustrated Review on Dental Autotransplantation Identifying Current Gaps in Knowledge

**DOI:** 10.3390/jcm14010017

**Published:** 2024-12-24

**Authors:** Akshay A. Chhana, Antonio J. Moretti, Adam D. Lietzan, John R. Christensen, Patricia A. Miguez

**Affiliations:** 1 Department of Periodontology, Endodontics and Dental Hygiene, Adams School of Dentistry, University of North Carolina at Chapel Hill, Chapel Hill, NC 27599, USA; 2 Department of Biomedical Sciences, Adams School of Dentistry, University of North Carolina at Chapel Hill, Chapel Hill, NC 27599, USA; 3 Department of Pediatric Dentistry and Dental Public Health, Adams School of Dentistry, University of North Carolina at Chapel Hill, Chapel Hill, NC 27599, USA

**Keywords:** tooth autotransplantation, dental trauma, periodontal ligament, pediatrics

## Abstract

**Background**: Permanently replacing missing teeth in the younger population is a clinical challenge. However, dental autotransplantation offers a viable treatment option in this demographic. To be performed predictably, it requires proper diagnoses, planning, and adherence to established guidelines in a multidisciplinary approach. Such guidelines should use evidence-based dentistry to anticipate and limit complications. The aim of this study is to evaluate the current literature on dental autotransplantation, discuss an evidence-based protocol, highlight steps for minimizing complications, and present a case report on two autotransplantations conducted at the Graduate Periodontology clinic at the University of North Carolina at Chapel Hill, Adams School of Dentistry, in the context of representative cases. **Methods:** A literature search using PubMed was conducted with the goal of constructing a comparative table on both survival and success rates of autotransplantation, providing a comparison of protocols and clinical studies and informing this narrative review. This review is illustrated by cases with the intention to highlight critical steps, limitations, and strengths of this mode of treatment. **Results and Conclusions:** Given the information presented, it is concluded that autotransplantation is a valuable treatment option provided established guidelines are followed. It is particularly useful for the younger population where implants or other options are not appropriate or feasible. This study also highlights some of the gaps in knowledge in autotransplantation which present opportunity for further studies to be developed.

## 1. Introduction

Dental autotransplantation (AT) is a surgical procedure involving the transplantation of a tooth from one location in the mouth to another within the same individual [1]. This procedure has gained attention in dentistry as an option for replacing missing or compromised teeth. The existing literature provides valuable insights into the biology, techniques, and outcomes associated with AT.

The concept of dental AT has its roots in early attempts to restore dentition. Historical records indicate rudimentary forms of tooth transplantation dating back to ancient Egypt [2]. However, modern dental AT has evolved significantly with advancements in surgical techniques, diagnostic tools, and our understanding of biology.

Indications for dental AT include agenesis, dental trauma, teeth with poor prognoses, and severely ectopically positioned teeth [3,4]. Contraindications include complex root morphology that complicate an atraumatic extraction, smoking, poor oral hygiene, or systemic conditions that could affect postoperative healing. Additionally, transplanting teeth with significant attachment loss or anatomical features such as enamel projections that could lead to attachment loss is not ideal [4].

During AT, the neural and vascular bundle of the pulp is severed. The donor tooth’s potential for revascularization in the recipient location depends on the stage of root development and, in particular, how open the root apices are [5,6]. The wider foramen allows for more capillary invasion, increasing blood supply [7]. If revascularization does not occur, pulp necrosis ensues. When the apical foramen is less than 1 mm, nonsurgical root canal therapy is recommended (preferably) before or 2 weeks after transplantation [7,8,9].

Regarding the periodontal ligament (PDL), its preservation during AT is of the utmost importance. Significant damage to the PDL can lead to ankylosis or inflammatory root resorption [7,10,11]. By orthodontically loading fully developed teeth prior to transplantation, the PDL will be enlarged, and the tooth will be easier to extract, lowering the chance of damaging the PDL during extraction [8,12].

Upon successful transplantation, histological studies show a vital PDL can be achieved [11,13]. As PDL cells can differentiate into cementoblasts, osteoblasts, and fibroblasts, the PDL of a transplanted tooth is capable of hard and soft tissue regeneration. Several studies show evidence of rapid bone formation, in addition to vertical bone and soft tissue augmentation, following AT. Complete periodontal healing is expected in 8 weeks [7,14,15,16].

Aside from AT, conventional options for replacing teeth include fixed appliances, orthodontic closure, and removable appliances [17]. Similar to implant therapy, AT acts as a fixed solution that does not require preparing virgin teeth. However, implant therapy is contraindicated in children and adolescents as they are still undergoing vertical skeletal growth [18]. Because an implant is not capable of eruption, placing one in a child would eventually result in an infraoccluded restoration. However, successfully autotransplanted teeth retain a vital PDL, therefore retaining the capacity for functional adaptation and continued alveolar bone remodeling. This offers a significant advantage over other treatment modalities when treating a younger population [8,16].

Definitions of success differ among AT studies. Common criteria for success included physiologic mobility, lack of inflammatory resorption, and continuation of root formation in immature teeth [8,16,19]. Survival rate is defined by the presence of the transplanted tooth in the mouth [19]. A recent systematic review and meta-analysis reports survival and success rates for teeth with an open apex as 98.21% and 89.68%, respectively [19]. While a systematic review and meta-analysis stratifying for success rates in teeth with complete root formation only could not be found, 1- and 5-year survival rates in this group were 98% and 90.5%, respectively [20]. In this article, we provide a narrative review on AT by discussing steps on minimizing complications and illustrating with detail the methodology of an evidence-based guideline via representative AT cases pursued at the Graduate Periodontology clinic at the University of North Carolina at Chapel Hill Adams School of Dentistry (UNC-CH) and discuss the protocols and gaps in knowledge in the context of modern dentistry.

## 2. Methods and Results

### 2.1. Literature Search

A literature search using PubMed was conducted with the goal of constructing a comparative table on survival and success rates, providing a comparison of protocols, and informing this critical narrative review. The reference lists found of the studies were hand-searched to identify the most relevant studies for protocol and outcomes comparison. The search was not limited to any specific dates or language (Table 1). After the literature search, the available AT protocols were compiled, along with the survival and success rates of various clinical studies as presented in Table 2 and Table 3. A librarian with extensive expertise in bibliographic search assisted the search and cross-checked findings with the main search conductor (A.A.C.). An independent reviewer (P.A.M.) resolved any divergence to eliminate the chance of bias during the search.

The search terms and criteria used are listed in Table 1.

### 2.2. Detailed Clinical Illustration

A 16-year-old male (medical history was not contributory, healthy, and patient was not on any medications) presented to the Graduate Periodontology clinic at UNC-CH with a missing maxillary right central incisor. The patient was involved in a motor vehicle accident where he sustained a fracture to the root. The tooth was then deemed nonrestorable and extracted by a different clinic two years prior.

The patient also presented with moderate mandibular crowding and moderate attachment loss (6 mm on mesial and buccal, Class II mobility using the Miller Index [21]) of tooth no. 7 [tooth no. 12 per FDI World Dental Federation notation (FDIn)] as a result of how the tooth no. 8 (FDIn no. 11) site healed. Orthodontically, the patient was classified as Class III growth pattern.

AT of tooth no. 29 (FDIn no. 45) → no. 8 (FDIn no. 11), extraction of tooth no. 7 (FDIn no. 12) and AT of no. 24 (FDIn no. 31) → no. 7 (FDIn no. 12), a Maryland bridge, and an acrylic partial were offered as treatment options to the patient. Orthodontic therapy was also recommended to the patient.

The patient, with consent from his parents, accepted the treatment plan consisting of orthodontic therapy, transplanting tooth no. 29 (FDIn no. 45) to the tooth no. 8 (FDIn no. 11) site, extracting tooth no. 7 (FDIn no. 12), and transplanting tooth no. 24 (FDIn no. 31) to the tooth no. 7 (FDIn no. 12) site. The UNC Trauma Team, composed of orthodontists, endodontists, periodontists, and pediatric dentists, believed this option would offer a high survival and success rate, address the mandibular crowding, and allow for functional adaptation and continued growth of the alveolar process. Additionally, autotransplanting tooth no. 24 (FDIn no. 31) to the site of the original tooth no. 7 (FDIn no. 12) would address the attachment loss while providing a more esthetic solution. Tooth no. 24 (FDIn no. 31) and tooth no. 29 (FDIn no. 45) were chosen as the donor teeth because their anatomy would allow for a more atraumatic extraction. The team assessment of the second premolar space led to the conclusion that it would be easier to close orthodontically due to anchorage, and their extraction would help address the mandibular crowding. The decision was made to perform each AT separately, allowing the team to address the specific needs of each procedure without the added difficulty of handling multiple complex surgeries simultaneously.

Consent for the surgeries and for including the case in this paper were signed by the patient’s legal guardian using a standardized electronic form at the Adams School of Dentistry at UNC-CH.

**Figure 1:**
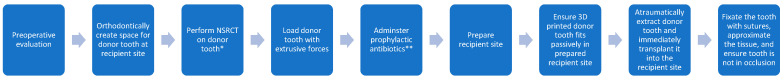
A short workflow was generated based on the protocol provided by Barendregt [8]. * Constricted apex only, ** open apex only. (NSRCT: nonsurgical root canal treatment).

The preoperative, surgical, and postoperative workflow was previously described by Andreasen and Barendregt [3,8]. A visual representation of this workflow is provided in Figure 1.

**Figure 2:**
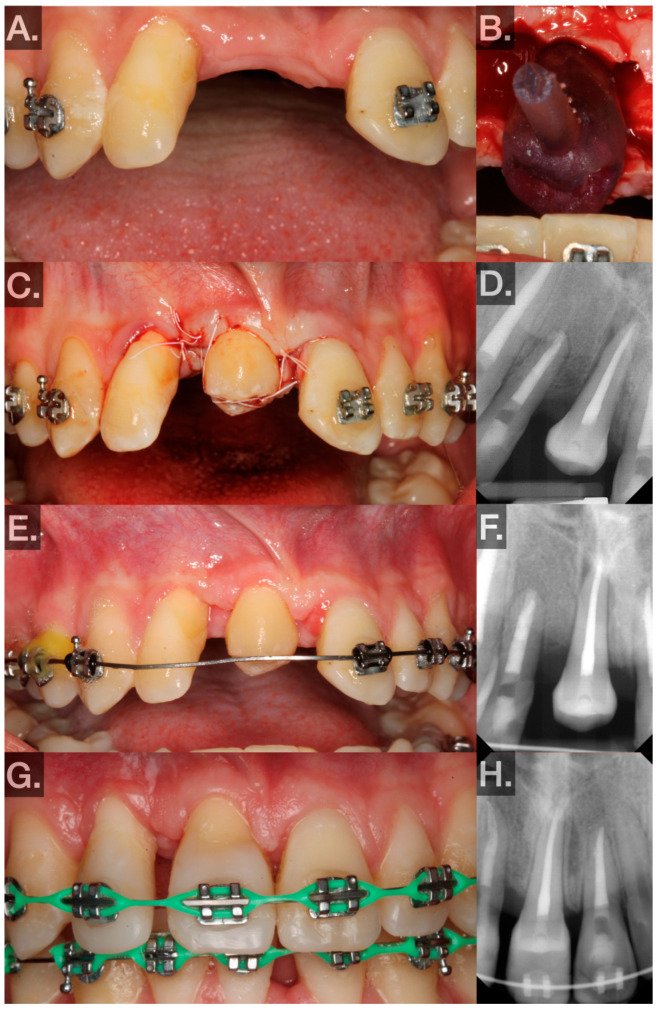
(**A**) Preoperative photo showing edentulous site no. 8 (FDIn no. 11). (**B**) Replica made by 3D printing of the donor tooth no. 29 (FDIn no. 45) in the artificially generated socket, verifying fit and passivity. (**C**) Transplanted no. 29 (FDIn no. 45) fixated subocclusally with sutures in the no. 8 (FDIn no. 11) site. (**D**) Immediate postoperative radiograph. (**E**) Six-week postoperative photo showing slight inflammation on the mesial of no. 9 (FDIn no. 21). (**F**) Six-week postoperative radiograph. (**G**) Ten-month postoperative view showing the transplant restored with resin composite to ideal contours. (**H**) Ten-month postoperative radiograph showing the root of the transplant surrounded by a lamina dura.

The preoperative phase was as follows:

○ Figure 2A illustrates the case as presented in the Graduate Clinic of Periodontology at UNC-CH. Because the roots of these teeth were fully developed, nonsurgical root canal therapy (NSRCT) was performed prior on tooth no. 24 (FDIn no. 31): 7 months preoperatively, 2/2023; tooth no. 29 (FDIn no. 45): 4 months preoperatively, 1/2023) to the AT. NSRCT was performed in a single visit by debriding canals to working length using K files and obturating with vertical technique. Donor teeth were orthodontically loaded with extrusive forces prior to each transplantation (tooth no. 24 (FDIn no. 31): 0.016 NiTi wire, 4 weeks preoperatively, 8/2023; tooth no. 29 (FDIn no. 45): 0.14 NiTi wire, 8 days preoperatively, 4/2023) to widen the PDL and facilitate a more atraumatic extraction. Brackets attached to the donor teeth were removed a few days prior to the surgery to provide the forceps better access during the surgery. Additionally, replicas of the donor teeth were segmented from cone beam computed tomography (CBCT) and 3D printed in P Pro Surgical Guide clear resin on a Straumann P40 (Straumann AG, Basel, Switzerland) to ensure adequate recipient site preparation and to limit extra-alveolar time of the donor teeth (Figure 2B). Using the same CBCT, measurements of the root length and root width at the cemento-enamel junction (CEJ) were made.

**Figure 3:**
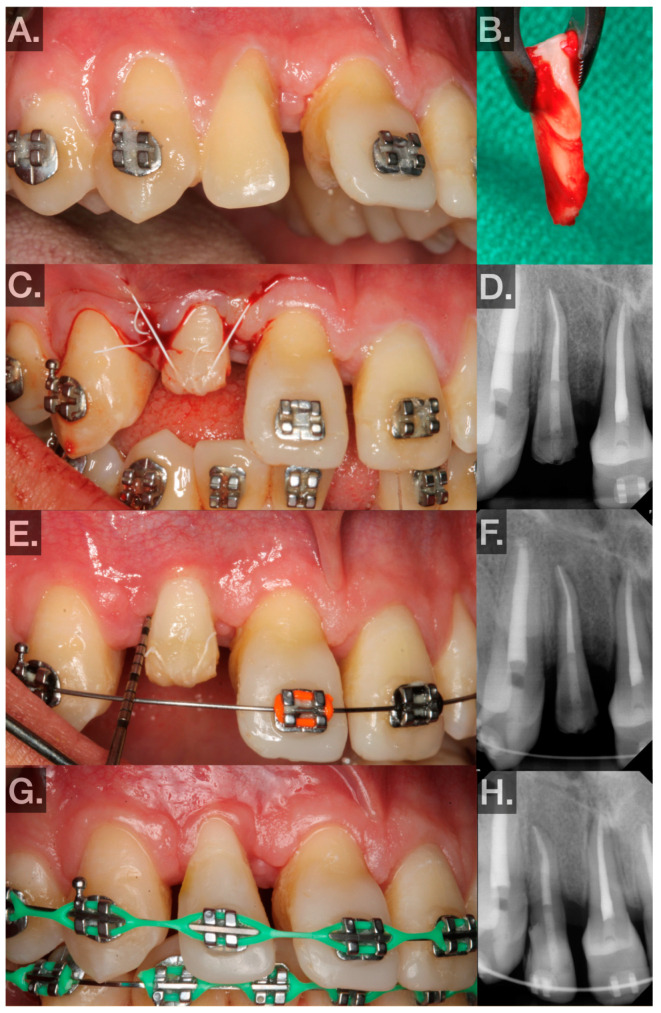
(**A**) Preoperative photo showing tooth no. 7 (FDIn no. 12) with mesial attachment loss. (**B**) Donor tooth no. 24 (FDIn no. 31) and its PDL after atraumatic extraction and before being transplanted. (**C**) Transplanted no. 24 (FDIn no. 31) fixated subocclusally with sutures in the no. 7 (FDIn no. 12) site. (**D**) Immediate postoperative radiograph. (**E**) Three-week postoperative photo showing the transplanted tooth gently probed with a UNC 15 probe. (**F**) Three-week postoperative radiograph. (**G**) Six-month postoperative photo showing the transplant restored to ideal contours. (**H**) Six-month postoperative radiograph.

The surgical phase was as follows:

○ Andreasen, Barendregt, and Louropoulou describe an antibiotic prophylaxis protocol [3,8,17]. Because teeth nos. 24 and 29 (FDIn nos. 31 and 45, respectively) had undergone NSRCT and had fully developed roots with a constricted apical foramen and because significant tissue inflammation was absent, antibiotics were not given prophylactically.
○ No. 29 (FDIn no. 45) → no. 8 (FDIn no. 11) (Figure 2A–H; 5/2023):
  ▪ Two carpules of Septocaine^®^ (articaine hydrochloride 4% and epinephrine 1:100,000; Septodont, Saint-Maur-des-Fossés, France) were used to anesthetize the donor tooth and recipient site through local infiltration. A midcrestal incision was made and a full-thickness flap was elevated in the tooth no. 8 (FDIn no. 11) area. The recipient site was then prepared by creating an osteotomy using an implant motor (Implant 900, Denstply Sirona, Charlotte, NC, USA) and implant drills (Bone Level Tapered Drills, Straumann AG, Basel, Switzerland.) The osteotomy was prepared to be slightly larger than the measurements made on the CBCT. Part of the buccal plate was intentionally obliterated in order to place the tooth in a proper buccolingual location. After the recipient site was prepared, the replica of the donor tooth was tried in (Figure 2B). Passive fit and subocclusal positioning were verified. Luxation forces were made with forceps on tooth no. 29 (FDIn no. 45) using slow rotational movements only. Special care was taken to ensure the beaks of the forceps did not seat apical to the CEJ. Tooth no. 29 (FDIn no. 45) was then extracted atraumatically and immediately placed into the artificially created socket at the site of tooth no. 8 (FDIn no. 11). The extra-alveolar time was less than 30 s. The tooth was fixated in the recipient site with 4–0 polytetrafluoroethylene (Cytosurg^®^ PTFE, Salvin, Charlotte, NC, USA) sutures via a Laurell–Gottlow suture technique [22]. Simple interrupted sutures were also used to approximate the tissue interproximally (Figure 2C). A periapical radiograph was then taken to serve as a baseline to evaluate healing (Figure 2D).
○ No. 24 (FDIn no. 31) → no. 7 (FDIn no. 12) (Figure 3A–H; 9/2023):
  ▪ First, 1.5 carpules of Septocaine® (articaine hydrochloride 4% and epinephrine 1:100,000; Septodont, Saint-Maur-des-Fossés, France) were used to anesthetize the donor tooth and recipient sites through local infiltration. Midcrestal and sulcular incisions were made and a full-thickness flap was elevated around tooth no. 7 (FDIn no. 12). The tooth was then extracted using elevators and forceps. The extraction socket was widened using an implant motor (Implant 900, Denstply Sirona, Charlotte, NC, USA) and implant drills (Bone Level Tapered Drills, Straumann AG, Basel, Switzerland). The osteotomy was prepared to be slightly larger than the measurements made on the CBCT. After the recipient site was prepared, the replica of the donor tooth was tried in. Passive fit and subocclusal positioning were verified. Luxation forces were made with forceps on tooth no. 24 (FDIn no. 31) using slow rotational movements only. Special care was taken to ensure the beaks of the forceps did not sit apical to the CEJ. Tooth no. 24 (FDIn no. 31) was extracted atraumatically and immediately placed into the artificially created socket after a photograph was taken to demonstrate the intact PDL (Figure 3B). The extra-alveolar time was less than 30 s. The tooth was fixated in the recipient site with 4–0 polytetrafluoroethylene (Cytosurg® PTFE, Salvin, Charlotte, NC, USA) sutures via a Laurell–Gottlow suture technique [22]. Because of the small occlusal surface area, composite resin (Filtek™ Supreme Flowable Restorative, 3M™, Saint Paul, MN, USA) was bonded to the tooth to hold the suture in place. Simple interrupted sutures were also used to approximate the tissue interproximally (Figure 3C). A periapical radiograph was then taken as a baseline for evaluating healing (Figure 3D).

The postoperative phase included the following:

○ The patient was instructed to rinse for 60 s twice a day with 0.12% chlorohexidine for 1 week. Additionally, the patient was instructed to take 400 mg ibuprofen and 325 mg acetaminophen every 4–6 h as needed for up to 5 days. Lastly, they were instructed to avoid strenuous activities for 1 week, avoid brushing the surgical area for 1 week, and avoid using the transplanted teeth for eating until after the 3-week postoperative visit.
○ Postoperative visits at 1, 3, and 6 weeks and 3, 6, and 12 months were planned.
  ▪ At 1 week, sutures were removed, and the patient was instructed to use a soft surgical toothbrush (Rx Ultra Suave, PHB Toothbrushes, Osseo, WI, USA) dipped in chlorohexidine twice daily to clean the clinical crown of the transplant for 2 weeks.
  ▪ At week 3, bleeding on probing (BOP), probing depth (PD), and mobility were measured at 6 sites to evaluate periodontal healing (Figure 3E). The patient was instructed to resume normal brushing and to use the tooth in normal function.
    - Periodontal findings: No measurements of PDs >4 mm were found, there was Class 1 mobility using the Miller Index [21], no suppuration, no BOP.
    - The 3-week postoperative photograph and radiograph for transplant no. 24 (FDIn no. 31) is shown in Figure 3E,F.
  ▪ At week 6, BOP, PD, and mobility were measured at 6 sites to evaluate periodontal healing.
    - Periodontal findings: No PDs > 4 mm were found, Class 1 mobility using the Miller Index [21], no suppuration, no BOP.
    - The 6-week postoperative photograph and radiograph for transplant no. 29 (FDIn no. 45) is shown in Figure 2E,F.
  ▪ Between weeks 6 and 8, the transplanted teeth were restored with composite resin (Filtek™ Supreme Ultra Universal Restorative, 3M™, Saint Paul, MN, USA) and orthodontically loaded with low-force 0.016 NiTi wires. Special care was taken at this point to avoid intrusive orthodontic forces.
  ▪ At every visit, oral hygiene instructions were reinforced, mobility was checked to rule out ankylosis, and periapical radiographs were taken to evaluate periodontal healing radiographically (Figure 2F,H and Figure 3F).
  ▪ Intraoral photographs and radiographs at the most recent postoperative visit (transplant no. 29 (FDIn no. 45): 10 months, transplant no. 24 (FDIn no. 31): 6 months) are illustrated in Figure 2G,H and Figure 3G,H.

**Table 2:** Reported survival and success rates from various studies across the literature on autotransplantation. * Immature teeth only, ** fully developed teeth only, *** fully developed teeth transplanted in adolescents only, **** teeth transplanted in adults only.

Study	Study Type	Observation Period	Donor Tooth Type	No. of Teeth/Studies	Survival Rate	Success Rate
Andreasen et al., 1990 [3,6]	Prospective study	1–13 years	Premolars	370 premolars	95% * 98% **	
Tsukiboshi 2002 [7]	Narrative review	Mean follow-up of 6 years	Mostly developed teeth	220 cases	90%	82%
Chung et al., 2014 [20]	Systematic review and meta-analysis	5 years	Teeth with completed root formation	26 studies	90.5% **	
Akhlef et al., 2017 [16]	Systematic review	9 months to 22 years, median 8.75 years	Premolars transplanted to anterior maxilla	11 studies	93% to 100%, weighted mean of 96.7%	
Atala-Acevedo et al., 2017 [19]	Systematic review and meta-analysis	Mean follow-up of 6 years	Teeth with open apex	21 studies	98.21% *	89.68% *
Jakobsen et al., 2018 [23]	Prospective study	Mean observation time was 10.1 years	Premolars	89 premolars transplanted: 54 by experienced surgeons, 35 by inexperienced surgeons	95%, no difference found between two surgeon groups	
Barendregt et al., 2023 [8]	Retrospective study	10 years	Premolars transplanted to the posterior region	1654 premolars	99.7% *, 95.7% *** 83.3% ****	99.4% *, 95.5% *** 83.3% ****
Louropoulou et al., 2023 [17]	Retrospective study	10 years	Premolars transplanted to the anterior region	910 premolars	99.8% *, 100% ***, 87.5% ****	99.8% *, 96.3% ***, 87.5% ****

**Table 3:** Compilation of protocols available in the literature for dental autotransplantation.

Scheme	Indications	Factors Contributing to Success	Timing of Root Canal Treatment	Timing of Orthodontic Movement	Antibiotics
Andreasen et al., 1990 [3,6,10]	Agenesis, trauma	Short extra-alveolar period, follow-ups that routinely consist of pulp testing and intraoral radiographs	4 weeks postoperatively	Not reported	Yes
Tsukiboshi 2002 [7]	Missing or hopeless teeth where a donor tooth can be used without causing harmful effects to its current position	Atraumatic extraction, tight closure of the gingival flap around the donor tooth	2 weeks after transplantation in mature teeth	1 month after transplantation in mature teeth	Yes
Barendregt et al., 2023 [8]	Agenesis, trauma, impacted or malformed teeth	Timely NSRCT treatment, timely orthodontic loading	6 weeks preoperatively	6–8 weeks postoperatively	Selectively
Louropoulou et al., 2023 [17]	Trauma, agenesis, impacted or malformed teeth	Timely NSRCT, orthodontically loading the donor tooth preoperatively and postoperatively, conservative restoration, strict postoperative follow-up	6 weeks preoperatively	6–8 weeks postoperatively	Selectively

## 3. Discussion

Data on survival and success rates from recent systematic reviews and meta-analyses were presented [19,20]. However, the results from the retrospective studies from Barendregt and Louropoulou are worth discussing specifically because of their large number of participants, high success rates, and high survival rates. Survival rate was defined by the presence of the transplanted tooth in the mouth. Criteria for success included continued root formation after transplantation, absence of deep periodontal pockets, normal tooth mobility, and effective treatment of root resorption or pulpal necrosis following transplantation. A total of 1654 premolars were transplanted to the posterior region in the Barendregt study, while 910 premolars were transplanted to the anterior region in the Louropoulou study. The 10-year success rates were 95.5% and 96.3%, respectively, in fully developed teeth transplanted in adolescents. In immature teeth, the 10-year success rates were 99.4% and 99.8%, respectively [8,17]. Their success and survival rates are higher than those reported by other studies. Barendregt suggests this might be due to the large study size, timely endodontic treatment, and timely orthodontic treatment (Table 2) [8].

Despite the high survival and success rates in AT, complications can still occur. Complications for AT include pulpal necrosis, inflammatory resorption, and ankylosis [3,6]. Pulpal necrosis occurs as a result of the autotransplanted tooth failing to revascularize and appears to be strongly related to the dimensions of the apical foramen. Moorrees et al. describes several stages of root development [5]. Barendregt and Andreasen took this classification and divided it into several stages. Stages 1–4 are one- to four-quarters of anticipated root length with an open apical foramen. Stage 5 is the fully developed root length with a half-open foramen. Stage 6 is a fully developed root length with a constricted apical foramen. Stage 7 is a fully developed root length with a closed apical foramen [3,5,8]. In a classical study by Andreasen where 370 premolars were autotransplanted, pulpal necrosis developed in 58 (15.7%) cases. Of these 58 cases, pulpal necrosis occurred in 100% of cases in Stage 6 root development, 60% of cases in Stage 5, and 4% of cases in Stages 0–4. Furthermore, 87% (26/30) of teeth transplanted with an apical diameter less than 1 mm preoperatively resulted in pulpal necrosis. In cases of pulpal necrosis, nonsurgical root canal therapy was performed 4 weeks after transplantation [6].

Inflammatory resorption presents as resorption concavities on the root surface typically accompanied with adjacent bone resorption and pulpal necrosis [6]. It is mostly associated with damage to the PDL, increased extra-alveolar time, and later stages of root development [6,7,8,11]. In Andreasen’s study, it occurred in 4.6% of cases. In all cases, resorption was halted by nonsurgical root canal therapy.

Ankylosis, or replacement resorption, is related to damage of more than 20% of the PDL and delayed orthodontic healing [7,8,11,12,17]. Czochrowska et al., Barendregt et al., and Louropoulou et al. reported rates of 4.4%, 4%, and 2.4%, respectively [8,17,24]. Andreasen, Barendregt, and Louropoulou successfully managed some cases of ankylosis by applying luxation forces surgically and orthodontically [8,10,17]. The suggested biological mechanism for this process is that the bony bridge between the root surface and alveolar bone is broken and replaced by a new fibrous tissue [17].

Complications can be minimized by strict adherence to the protocol provided by Barendregt et al. and by coordinating with a team consisting of multiple specialties [8]. Pulpal necrosis and inflammatory resorption rates can be minimized by performing nonsurgical root canal therapy (NSRCT) in late Stage 6 or 7 root development, where the apical foramen is less than 1 mm, 6 weeks before or 2 weeks after AT [7,8]. The rationale for NSRCT preoperatively is that endodontic therapy is more successful in vital pulp than when necrotic [8]. Orthodontically preloading Stage 6 and Stage 7 teeth will minimize damage to the PDL by enhancing tooth mobility and facilitating a more atraumatic extraction, thereby reducing the risk of ankylosis [8,12]. Using software to segment the donor tooth from a CBCT and printing it at a size of 110% prior to the surgery is recommended. This will ensure that the artificial socket is satisfactory prior to extraction, minimizing the extra-alveolar time of the transplant. The 10% increase in size accommodates the PDL. Anterior teeth should be restored with a composite veneer at 6 weeks and loaded orthodontically no later than 8 weeks after surgical therapy [8]. Care should be made to not prepare into dentin or remove any palatal cusps, particularly in developing teeth. Doing so could compromise pulp vitality by creating a route for bacterial invasion through exposure of dentinal tubules [25]. Additionally, restoring the tooth to ideal esthetics at 6 weeks will aid the orthodontist in planning movement forces [8,17]. Lastly, where appropriate, antibiotics can help minimize complications [20]. Louropoulou showed that the incidence of endodontic complications can be reduced from 14.7% to 3.3% by prescribing antibiotics prophylactically (3 mg amoxicillin, 1 h before) when transplanting vital teeth with a Moorrees’ Stage 4, 5, or 6 root development. One explanation of how antibiotics may help is by reducing the ingress of bacteria to a vulnerable pulp. The antibiotics help protect the vulnerable pulp from intraoral and extraoral sources of bacteria [20]. In cases where inflammation is present at the recipient site, Barendregt recommends prescribing amoxicillin and metronidazole (dosage depends on the weight of the patient) three times daily for 7 days starting 1 day before the surgery. When the patient has a penicillin allergy, clindamycin is recommended as an alternative [8]. Because the donor teeth in this case were late Stage 6, NSRCT was performed prior to treatment and no antibiotics were prescribed.

Although not a true complication, lack of root development in immature teeth following AT should be considered. If preserved, Hertwig’s epithelial root sheath will retain its growth capacity after AT and continued root development can be expected [7,26]. However, transplanted immature teeth are on average shorter than their contralateral teeth [26,27]. Root development can be expected to be complete in 21%, partially complete in 65%, and arrested in 14% of transplanted teeth [27]. Given this information, it would be ideal to transplant teeth where root development is near complete but the apex is >1 mm. Much of the literature on AT states that the ideal time to perform AT is around Stage 3 [16,28]. However, Barendregt, who reports a very high success and survival rate in 1654 premolars, states that the most ideal time is from Stage 4 to the beginning of Stage 5 of root development [8].

Splinting helps provide initial stability to the transplanted tooth during healing. However, different splinting materials and durations can alter healing [20]. A study on autotransplanted teeth in monkeys showed that rigid splinting actually had a negative impact on periodontal healing [29]. Alternatively, splinting with sutures allows for a small amount of physiologic movement and loading, which can reduce complications such as ankylosis [4,20,30]. A study by Bauss et al. compared rigid splinting with a wire for four weeks versus suture splinting for one week in 76 transplanted third molars and a mean follow-up of 3.4 years. In the study, success was defined as the tooth being present without signs of inflammatory root resorption, ankylosis, pathological pockets, abnormal mobility, or periapical radiolucencies. They reported a 93% success rate for the teeth stabilized with sutures and a 73.5% success rate for the teeth splinted with a composite bonded wire. They reported that increased rates in ankylosis and pulpal necrosis were to blame for the difference in results [30].

While implants dominate the private practice setting, AT offers similarities, advantages, and disadvantages. As mentioned earlier, autotransplanted teeth are capable of functional adaptation. This offers a significant advantage over implants as they can be placed in a younger population and can be moved orthodontically after transplantation [16]. AT is also more cost-effective for the patient [7,8]. In a private practice setting, one oral surgery office in New Hampshire reports that autotransplants cost 87% less than a dental implant in their office [31]. Regarding esthetics, patient reported satisfaction regarding their outcomes of autotransplanted teeth in the anterior maxilla, ranging from 82% to 100% [16]. A study on 96 implants placed in the anterior maxilla reported a 96% patient satisfaction rate [32]. Although a study with a direct comparison is needed, Akhlef et al. call attention to how the patient-reported outcomes of these two groups are comparable [16]. While promising, the AT patient satisfaction studies have small sample sizes, and more research is warranted before conclusions can be made. A major disadvantage of AT is that it requires an adequate donor tooth whose transplantation would not cause harmful effects to its original position [7,31]. Additionally, adequate space (1 mm of clearance from neighboring teeth post-transplantation) at the recipient site is required [3]. Orthodontic therapy could help create space preoperatively or close the space of the original donor tooth postoperatively. However, if the patient does not have any other orthodontic needs, it might be worth considering other options.

After a thorough review of the literature, a few gaps in knowledge have been identified. With no uniformity in criteria for success, it is difficult to interpret the success rates presented in meta-analyses. Additionally, there is a lack of randomized control trials to compare healing. Thus, one limitation of our search is that confounders could not be accurately identified and controlled for. Lastly, the private practice application and cost analyses of AT in the United States are yet to be seen and were not able to be assessed via the literature search. While the interdisciplinary and collaborative approach that this treatment option requires may be difficult, routinely extracted third molars are viable donor teeth. Their role in the private practice setting could be explored as an option to making this procedure more frequent. After interviewing 20 specialists, a study by Dokova et al. identified domains that explained the barriers affecting the adoption of AT in private practice. Collaboration between peers and the cultural framework of the United States healthcare system were two of the reported domains [33]. However, only pediatric dentists and orthodontists were interviewed in this study. Further studies are needed to assess the perspectives of periodontists and oral surgeons regarding AT in a private practice setting.

Lastly, in the clinical case presented, radiographic and clinical follow-ups have not shown complications such as root resorption and ankylosis in the initial follow-ups. The patient and parents were aware that if the AT brings such complications in later stages, these complications are manageable, leading to successful remediation, and the outcome is still advantageous given the preservation of the alveolar bone and gingival health. In the future, an AT patient is ultimately a good candidate for dental implant treatment and/or prosthesis, which are not the recommended treatments for children and adolescents. Another advantage of AT is a long-term satisfactory esthetic outcome for young adults while waiting for complete alveolar bone growth. Regarding the case reports, it is a limitation of this study that long-term follow-up of the cases has not been possible due to the discontinuation of this patient’s care at the graduate clinic.

## 4. Conclusions

Given the information in this article and the case presented, AT is a valuable treatment option for patients. It is particularly useful in younger patients where implants or other options are not appropriate. By utilizing a multidisciplinary approach and following a strict guideline, AT can be performed successfully and predictably.

## Figures and Tables

**Table 1:** Manuscript selection process.

Search Tool + Criteria	Results	Terms
PubMed—systematic, scoping, umbrella Reviews, and meta-analyses	33	((dental autotransplantation[tw] OR tooth autotransplantation[tw] OR teeth autotransplantation[tw] OR autogenous tooth transplantation[tw] OR autogenous teeth transplantation[tw] OR “autotransplantation of teeth”[tw] OR ((autotransplantation[tw] OR auto-transplant*[tw] OR autotransplant*[tw] OR “Transplantation, Autologous”[Mesh] OR autologous transplant*[tw]) AND (dental[tiab] OR dentistry[tiab] OR teeth[tiab] OR tooth[tiab] OR periodontics[tiab] OR periodontology[tiab] OR periodontal[tiab] OR molar*[tiab] OR premolar*[tiab] OR canine[tiab] OR canines[tiab]))) NOT ((“animals”[mesh] NOT “humans”[mesh]))) AND (systematic[tw] OR scoping[tw] OR systematized[tw] OR umbrella[tw] OR meta-analyses[tw] OR meta-analysis[tw])
PubMed—randomized clinical trials	36	((dental autotransplantation[tw] OR tooth autotransplantation[tw] OR teeth autotransplantation[tw] OR autogenous tooth transplantation[tw] OR autogenous teeth transplantation[tw] OR “autotransplantation of teeth”[tw] OR ((autotransplantation[tw] OR auto-transplant*[tw] OR autotransplant*[tw] OR “Transplantation, Autologous”[Mesh] OR autologous transplant*[tw]) AND (dental[tiab] OR dentistry[tiab] OR teeth[tiab] OR tooth[tiab] OR periodontics[tiab] OR periodontology[tiab] OR periodontal[tiab] OR molar*[tiab] OR premolar*[tiab] OR canine[tiab] OR canines[tiab]))) NOT ((“animals”[mesh] NOT “humans”[mesh]))) AND (randomizedcontrolledtrial[Filter])
PubMed—clinical trials	104	((dental autotransplantation[tw] OR tooth autotransplantation[tw] OR teeth autotransplantation[tw] OR autogenous tooth transplantation[tw] OR autogenous teeth transplantation[tw] OR “autotransplantation of teeth”[tw] OR ((autotransplantation[tw] OR auto-transplant*[tw] OR autotransplant*[tw] OR “Transplantation, Autologous”[Mesh] OR autologous transplant*[tw]) AND (dental[tiab] OR dentistry[tiab] OR teeth[tiab] OR tooth[tiab] OR periodontics[tiab] OR periodontology[tiab] OR periodontal[tiab] OR molar*[tiab] OR premolar*[tiab] OR canine[tiab] OR canines[tiab]))) NOT ((“animals”[mesh] NOT “humans”[mesh]))) AND (random*[tiab] OR prospectiv*[tiab] OR (clinical[tiab] AND trial[tiab]))
PubMed—protocols	98	(((dental autotransplantation[tw] OR tooth autotransplantation[tw] OR teeth autotransplantation[tw] OR autogenous tooth transplantation[tw] OR autogenous teeth transplantation[tw] OR “autotransplantation of teeth”[tw] OR ((autotransplantation[tw] OR auto-transplant*[tw] OR autotransplant*[tw] OR “Transplantation, Autologous”[Mesh] OR autologous transplant*[tw]) AND (dental[tiab] OR dentistry[tiab] OR teeth[tiab] OR tooth[tiab] OR periodontics[tiab] OR periodontology[tiab] OR periodontal[tiab] OR molar*[tiab] OR premolar*[tiab] OR canine[tiab] OR canines[tiab]))) NOT ((“animals”[mesh] NOT “humans”[mesh]))) AND (practiceguideline[Filter] OR protocol[tw] OR protocols[tw] OR guideline[tw] OR guidelines[tw] OR consensus[tw] OR statement[tw] OR statements[tw] OR guidelines as topic[MeSH:noexp] OR clinical protocols[MeSH:noexp] OR Consensus[MeSH:noexp]))
